# Chemical composition of polyphenols extracted from strawberry pomace and their effect on physiological properties of diets supplemented with different types of dietary fibre in rats

**DOI:** 10.1007/s00394-013-0557-z

**Published:** 2013-07-12

**Authors:** Monika Kosmala, Zenon Zduńczyk, Krzysztof Kołodziejczyk, Elżbieta Klimczak, Jerzy Juśkiewicz, Przemysław Zduńczyk

**Affiliations:** 1 Institute of Chemical Technology of Food, Lodz University of Technology, ul. B. Stefanowskiego 4/10, 90-924 Lodz, Poland; 2 Division of Food Science, Institute of Animal Reproduction and Food Research, Polish Academy of Sciences, ul. Tuwima 10, 10-747 Olsztyn, Poland

**Keywords:** Ellagitannins, Ellagic acid, Proanthocyanidins, Strawberry pomace

## Abstract

**Purpose:**

The objective of this study was to establish the composition of polyphenolic preparations obtained from industrial strawberry pomace with two methods of extraction: the water and the water-alcoholic one and then to analyse their effects in the gastrointestinal tract depending on the composition of dietary fibre—cellulose or fructooligosaccharides (FOS).

**Methods:**

Freeze-dried water extract (PTW), containing 5.1 % of ellagic acid, 0.2 % of proanthocyanidins, and soluble carbohydrates as a major part, and water–alcohol extract (PTE), containing 17.1 % of ellagic acid and 10.9 % of proanthocyanidins, were administered, in the equivalent quantity of 0.06 % of ellagic acid, to 4- to 8-week-old rats (8 animals per group), as a component of modified AIN-93 diets containing 5 % of cellulose or FOS.

**Results:**

The addition of strawberry pomace extracts had no effect on either the diet intake or the body weight of experimental rats. Both extracts, similarly to FOS, beneficially reduced the activity of β-glucuronidase in caecal digesta, with the PTW effect being significantly higher than that of PTE (7.59 vs. 9.20 μmol/h/g, *P* = 0.001). In comparison with PTE, the PTW extract significantly increased the caecal digesta mass (1.45 vs. 1.27 k/kg BW) and the total production of SCFA (86.1 vs. 71.4 μmol/100 g BW). The extract enhanced the physiological effect of FOS by inhibiting the activity of β-glucuronidase, increasing the caecal digesta mass and SCFA production. Such an effect was not recorded in the case of the PTE preparation.

**Conclusions:**

The addition of strawberry pomace extracts affected the activity of certain enzymes of intestinal microflora and its most important products.

## Introduction

One of the important elements of health prophylaxis in economically developed countries is the increase in the consumption of vegetables and fruit being the source of biologically active dietary components that have a beneficial influence on human health [1]. An additional source of these compounds in diet might be by-products of plant food processing [2], including fruit pomace, e.g. of strawberry.

In a number of countries, the harvest season of strawberry is short and during the industrial production of juices a considerable amount of biologically active components remains in fruit pomace. This still underestimated by-product may constitute a source of health-promoting substances, particularly dietary fibre and polyphenols [3, 4]. One of the possibilities is to use strawberry seeds (achenes) in the production of gluten-free bread with an increased content of dietary fibre and polyphenols [5]. Another possibility, particularly advisable in the case of an increased content of sand and physical impurities in pomace, is the extraction of biologically active components, polyphenols in particular [6]. Due to the application of enzymes degrading polysaccharides of the cell walls of the processed fruit, the composition of polyphenols may differ from that of fruit, and these compounds may be more easily extracted with various solvents [7, 8].

Strawberry polyphenols, mainly ellagitannins and ellagic acid, demonstrate a wide spectrum of positive effects on human health [9], primarily due to antioxidant [10, 11], anti-carcinogenic [12], anti-inflammatory and anti-neurogenerative [13, 14] effects. Such properties are exhibited by polyphenol metabolites present in blood after the digestion process of polyphenols as well as after their metabolism in the gastrointestinal tract and liver [15].

Products of ellagitannins hydrolysis and metabolism in the gastrointestinal tract are derivatives of hydroxy-6H-dibenzopyran-6-one defined as urolithin A and B, which are glucuronidated and sulphated in the intestinal-liver circulation, then participate in the metabolic process and are also excreted with faeces and urine [12, 16, 17]. Results of a research on ellagitannins and ellagic acid bioavailability and metabolism prove that these substances and their derivatives might occur in the digestive system in considerable concentrations as products of digestion with own enzymes, particularly with these of the large intestine [16]. Metabolism of ellagitannins is dependent on individual colonic microflora, high or low production of urolithins can occur [18]. Different urolithin hydroxylation patterns were observed for different animal species, suggesting that the microbiota responsible for the metabolism of ellagitannins in each animal species produces dehydroxylases for the removal of specific hydroxyls from the ellagic acid residue [19]. Glucuronide and sulphatic derivatives (sulphates) of urolithins transported with blood may have a beneficial effect on the level and proportions of cholesterol fractions [20, 21], may decrease the blood level of lipids [22] and influence remote organs [21] and have effects in the vascular inflammation [23].

Biodiversity and physiological effects of polyphenols are known to depend on their solubility determined by the chemical structure and the size of molecules as well as by links with other compounds, mainly fibre [13]. Diversified intensiveness of the fermentation processes in the intestines, determined by the composition and physiochemical properties of dietary fibre, might constitute an additional factor in this respect [24]. In this context, interesting from both cognitive and practical perspective are the two following issues: what is the composition of preparations of polyphenols that may be obtained from strawberry pomace with simple extraction methods, and what is the effect of the content and properties of dietary fibre on the physiological properties of these compounds.

The objective of the presented study was to examine the composition of water and water–ethanol extracts obtained from industrial strawberry pomace as well as to broaden knowledge on the physiological effects of polyphenols of these extracts in the gastrointestinal tract depending on the composition of dietary fibre, i.e. contents of sparingly fermentable cellulose and easily fermentable fructooligosaccharides.

## Materials and methods

### Materials

Water and ethanol strawberry extracts were obtained from industrial strawberry pomace produced on a modern production line for concentrated fruit juices at the ALPEX Company (Łęczeszyce, Poland). To this end, 10 kg of fresh pomace collected from the production line was dried in a convection dryer (1.6 kW KC-100/200, WAMiE, Warsaw, Poland) at the temperature 65–70 °C for 8 h. The dried material (4.9 kg) was passed through sieves with 2- and 5-mm mesh diameter using an Analysette 3 sifter (Fritsch, Idar-Obersten, Germany). Fractions with particle sizes over 5 mm were ground on a universal grinding machine NMK-110 (Spomasz, Nakło, Poland); next, the grounded material was once more passed through two sieves. The material with particle sizes between 2 and 5 mm and the weight of 1.66 kg constituted part of pomace devoid of seeds (achenes) and was used for successive production of water and ethanol extracts applied in the present study. The composition of pomace (seedless 2–5 mm fraction) on dry matter basis was as follows: total carbohydrates 69 %, protein 21 %, ash 8 %, including 6 % sand; fat 5.5 %, polyphenols 3.9 %, including 2.4 % proanthocyanidins; 1.5 % free ellagic acid and its derivatives in the form of ellagitannins; 0.11 % flavonols and 0.09 % anthocyanins. The water extract was produced with the use of 1,500 g of dried material with particle size between 2 and 5 mm. Water extraction was conducted three times at the temperature of 65–70 °C for 1 h, at the solvent to material ratio of 4:1. Next, the extract was each time separated from the pomace in a laboratory screw press with the basket volume of 4 L. The resultant extract was condensed in a vacuum evaporator and next dried in a dryer under the pressure of 40 mm Hg, at the temperature of 70 °C. The yield of dry water extract (PTW) production amounted to 7.2 % per seedless strawberry pomace.

In order to obtain the water–alcohol extract, 4 L of 90 % ethanol was added to 3.5 kg of water-extracted pomace with water content of 59 %, which enabled obtaining the solvent to extraction material ratio of 4:1 and 60 % concentration of ethanol in the extractant. After 24 h of extraction at the temperature of 20 °C, the extract was separated from pomace with the laboratory press. Once more, 3.5 L of 60 % ethanol was added to the pomace, and the sample was left for 24 h. Next, the extract was pressed in the laboratory press. Ethanol was expelled from the two combined extracts. Afterwards, the solution was subject to freeze-drying in a TG 5 lyophilizer (VEB Hochvakuum Dresden, Germany), which enabled obtaining a dry extract of strawberry polyphenols. The yield of ethanol extract (PTE) production amounted to 3.1 % per dry seedless strawberry pomace.

### Determination of the composition of strawberry polyphenolic preparations

#### Basic composition

Basic composition of extracts was determined with the AOAC methods [25] using the following procedures: protein content—920.152; crude fat—930.09; dry matter and ash content—940.26, total dietary fibre (TDF)—985.29, insoluble dietary fibre (IDF) 991.42, respectively. Soluble dietary fibre was calculated as a difference between TDF and IDF. Simple sugars, sorbitol and sucrose were determined as described previously [4] by boiling extraction with 0.02 mol/L CaCO_3_, filtration and desalination in an anion exchange column (two parts of Amberlite IRA 400 anion exchanger to one part of Amberlite IR 120 cation exchanger) and analysis in a Knauer HPLC set (Berlin, Germany) with an RI detector and a Bio-Rad Aminex HPX-87C column, 300 × 7.8 mm (Phenomenex, Torrance, CA, USA), with the flow rate of 0.5 mL/min, at the temperature of 85 °C. Glucose, fructose, sucrose and sorbitol (Sigma-Aldrich, St Louis, MO, USA) were used as standards.

#### Determination of polyphenols with the HPLC method

The extraction was conducted as described previously [4], 3 times with 70 % methanol in ultrasounds (15 min). The extract was analysed with the HPLC method in an HPLC system with a DAD detector (Dionex, Sunnyvale, CA, USA) and with 250 × 4.60 mm Gemini 5u C18 110A column (Phenomenex, Torrance, CA, USA). Phase A consisted of 0.05 % phosphoric acid in water, whereas phase B of 0.05 % phosphoric acid in acetonitrile. The following gradient was applied at the flow rate of 1.25 mL/min: 5-min stabilization with 4 % B, next from 5 to 12.50 min with 4–15 % B, from 12.50 to 42.40 min with 15–40 % B, from 42.40 to 51.80 min with 40–50 % B, from 51.80 to 53.40 min with 50 % B and from 53.40 to 55 min with 4 % B. The column’s temperature was set at 25 °C.

#### Identification of polyphenols of strawberry preparations

The following standards were used for identification: ellagic acid (Extrasynthese, Genay, France), quercetin glucoside (quercetin-3-O-glucopyranoside, Extrasynthese, Genay, France), quercetin galactoside (Extrasynthese, Genay, France), kaempferol glucoside (kaempferol-3-O-glucoside, Extrasynthese, Genay, France), quercetin rhamnoside (Extrasynthese, Genay, France), quercetin (Extrasynthese, Genay, France) and kaempferol (Extrasynthese, Genay, France), at 360-nm wavelength.

Pelargonidin glucoside (Extrasynthese, Genay, France) was determined at the wavelength of 520 nm. The content of catechin and derivatives of coumaric acid were determined based on the measurement of UV-DAD spectrum at 280 nm and the application of the following standards: catechin (Sigma-Aldrich, St Louis, USA) and p-coumaric acid (Sigma-Aldrich, St Louis, USA). The content of ellagitannins was determined based on the amount of ellagic acid released in standard conditions of acidic hydrolysis [6].

#### Analysis of proanthocyanidins and free catechins

Proanthocyanidins of the extracts were determined from lyophilized samples. Proanthocyanidins degradation method in an acidic environment with an overdose of phloroglucinol was used. The method involving the use of phloroglucinolysis reaction was described by Kennedy and Jones [26]. About 20 mg of a powdered sample (pomace or extract) was weighed to a 2-mL Eppendorf tube, and then, 800 μL of a methanol solution containing phloroglucinol (75 g/L) and ascorbic acid (15 g/L) was added to the sample. Phloroglucinolysis reaction was begun by adding 400 μL of 0.2 M hydrochloric acid in methanol. The incubation was carried out for 30 min at the temperature of 50 °C. Next, the samples were instantly cooled down in a bath with ice, and the reaction was stopped by adding 600 μL of a 40 mM sodium acetate solution. The samples were next centrifuged for 5 min at 3,600*g* and then diluted twice with a 40 mM sodium acetate solution. Before being injected onto the HPLC system, the sample was stored at the temperature of 4 °C. Products of acidic degradation of polymeric proanthocyanidins were separated with the Knauer Smartline chromatograph (Berlin, Germany) equipped with a UV–Vis P2800 detector (Knauer, Berlin, Germany) and a fluorescent detector (FD) RF-10AXL (Schimadzu, Tokyo, Japan). The separation was conducted on Gemini 5u C18 110A 250 mm × 4.6 mm, 5 μm column with gradient elution with 2.5 % water solution (v/v) of acetic acid (phase A) and 80 % (v/v) acetonitrile in water (phase B). The following gradient was used: 0–10 min, 4–7 % B; 10–27 min, 7–30 % B; 27–29 min, 30–70 % B; 29–34 min, 70 % B; 34–35 min, 70–4 % B; and 35–40 min, 4 % B. The flow rate amounted to 1 mL/min, the temperature of separation to 25 °C and the volume of injection to 20 μL. The chromatographic data were collected with the use of ClarityChrom programme (Knauer, Berlin, Germany). The identification of components was conducted on the basis of comparing the retention times and UV–Vis spectra of standards of (−)-epicatechin, (+)-catechin, (−)-epigallocatechin, adducts: (−)-epigallocatechin-phloroglucinol, (−)-epicatechin-phloroglucinol and (+)-catechin-phloroglucinol. Quantitative analyses of released flavonols, i.e. (+)-catechin and (−)-epicatechin, were conducted on the basis of chromatograms recorded with the FD detector set at 278-nm excitation wavelength and 360-nm emission wavelength. Phloroglucinol adducts were determined on the basis of chromatograms registered with the PDA detector set at 280-nm wavelength. In order to calculate the released ellagitannins, use was made of calibration curves determined for (−)-epicatechin and (+)-catechin. In turn, a calibration curve determined for (−)-epicatechin-phloroglucinol adduct was used for adducts. Free (+)-catechin, (−)-epicatechin and (−)-epigallocatechin were determined from extracts as described above.

### Rats and diets

The use of animals was conducted in compliance with European guidelines for the care and use of laboratory animals and was approved by the Ethical Committee for Animal Experiments in the northeast Poland region. The experiment was performed on 48 male Wistar rats aged approximately 4 weeks. The experimental diets were administered for 4 weeks to 8 rats per group housed individually in plexiglas cages.

Diets contained similar contents of protein (from casein supplemented with methionine), fat (soybean oil), minerals and vitamins (from AIN-93G mixtures [27] and a similar content of fibre, however, from different sources, i.e. cellulose or fructooligosaccharides (Table 1). The compared polyphenolic preparations were administered in an amount of 1.13 % (PTW_C_ and PTW_FOS_) and 0.37 % (PTE_C_ and PTE_FOS_) of the feed air-dried, which constitutes 0.06 % of the total ellagic acid in a diet as the basic active substance of strawberries according to the current state of knowledge [12, 13, 21]. Experimental diets and tap water were administered ad libitum. The animals were maintained under standard conditions: temperature of 21–22 °C and relative air humidity of 50–70 %, intensive ventilation of rooms (15 × /h) and 12-h lighting. Individual body weights and food intakes were recorded.

**Table 1:** Composition of experimental diets, %

Component	Experimental group					
	C_C_	C_FOS_	PTW_C_	PTW_FOS_	PTE_C_	PTE_FOS_
Casein	14.0	14.0	14.0	14.0	14.0	14.0
Methionine	0.2	0.2	0.2	0.2	0.2	0.2
Soybean protein	1.9	1.9	1.77	1.77	1.83	1.83
Soybean oil	8.0	8.0	8.0	8.0	8.0	8.0
Cellulose	5.0	–	5.0	–	5.0	–
FOS	–	5.0	–	5.0	–	5.0
Extract PTW^a^	–	–	1.13	1.13	–	–
Extract PTE^b^	–	–	–	–	0.37	0.37
Cholesterol	0.5	0.5	0.5	0.5	0.50	0.5
Minerals^c^	3.5	3.5	3.5	3.5	3.50	3.5
Vitamins^d^	1.0	1.0	1.0	1.0	1.00	1.0
Fructose	60.0	60.0	60.0	60.0	60.00	60.0
Maize starch	5.9	5.9	5.9	5.9	5.6	5.6

^a^Strawberry polyphenols extracted with water

^b^Strawberry polyphenols extracted with ethanol

^c^AIN-93G [27], per kg mix, g: calcium carbonate anhydrous (40.04 % Ca) 357, potassium phosphate monobasic (22.76 % P, 28.73 % K) 196, potassium citrate and tripotassium monohydrate (36.16 % K) 70.78, sodium chloride (39.34 % Na, 60.66 % Cl) 74, potassium sulphate (44.87 % K, 18.39 % S) 46.6, magnesium oxide (60.32 % Mg) 24, ferric citrate (16.5 % Fe) 6.06, zinc carbonate (52.14 % Zn) 1.65, sodium meta-silicate × 9H_2_O (9.88 % Si) 1.45, manganous carbonate (47.79 % Mn) 0.63, cupric carbonate (57.47 % Cu) 0.3, powdered sucrose 221.026, chromium potassium sulphate × 12H_2_O (10.42 % Cr) 0.275; mg: boric acid (17.5 % B) 81.5, sodium fluoride (45.24 % F) 63.5, nickel carbonate (45 % Ni) 31.8, lithium chloride (16.38 % Li) 17.4, sodium selenate anhydrous (41.79 % Se) 10.25, potassium iodate (59.3 % I) 10, ammonium paramolybdate × 4H_2_O (54.34 % Mo) 7.95, ammonium vanadate (43.55 % V) 6.6

^d^AIN-93G [27], g/kg mix: nicotinic acid 3.0, Ca pantothenate 1.6, pyridoxine–HCl 0.7, thiamin-HCl 0.6, powdered sucrose 974.655, riboflavin 0.6, folic acid 0.2, biotin 0.02, vit. B_12_ (cyanocobalamin, 0.1 % in mannitol) 2.5; IU/g: vit. E (all-rac-α-tocopheryl acetate, 500) 15.0, vit. A (all-trans-retinyl palmitate, 500000) 0.8, vit. D_3_ (cholecalciferol, 400000) 0.25, vit. K-1 (phylloquinone) 0.075

#### Sample collection and analysis

During 4 weeks of experiment, faeces were collected and frozen at −70 °C for microbial enzymes activity determination. The microbial enzymes (β-glucosidase and β-glucuronidase) activity was measured by the rate of p- or o-nitrophenol release from their nitrophenyl-glucosides and expressed as μmol of product formed per minute (1 μmol/min = 1 Unit) per gram of caecal digesta [28].

After 4 weeks, the rats were anaesthetized using sodium pentobarbitone. Blood samples were taken from aorta. Serum was prepared by centrifugation at 1,500*g* for 15 min at 4 °C and stored at −40 °C until analysed. After laparotomy, the selected parts of the digestive tract (small intestine, caecum and colon) were removed and weighed. As soon as possible after euthanasia (ca. 10 min), ileal, caecal and colonic pH were measured, sample of digesta was taken to determine dry matter, ammonia and SCFA and the rest were frozen at −70 °C for microbial enzymes activity determination. The ileal, caecal and colonic walls were flushed clean with ice-cold saline, blotted on filter paper and weighed as the tissue weight.

The caecal pH was measured using a microelectrode and a pH/ION metre (model 301, Hanna Instruments, Vila do Conde, Portugal). Dry matter of fresh digesta was measured at 105 °C. Ammonia was extracted and trapped in a solution of boric acid, then determined by direct titration with sulphuric acid [29]. The amounts of SCFA were measured using gas chromatography under conditions described previously [28]. The SCFA pool size in a caecum was calculated as the product of SCFA concentration and caecal digesta mass. The microbial enzymes (α- and β-glucosidase, α- and β-galactosidase and β-glucuronidase) activity was measured by the rate of p- or o-nitrophenol release from their nitrophenyl-glucosides and expressed as μmol of product formed per minute (1 μmol/min = 1 Unit) per gram of caecal digesta [28].

All results obtained were worked out statistically using a two-way analysis of variance and the Duncan’s multiple range test at a significance level of *P* ≤ 0.05 using STATISTICA 6.0 (StatSoft Corp., Kraków, Poland) software.

## Results

### Composition of polyphenolic preparations

The PTW preparation was characterized by a significant content of dietary fibre in the form of soluble fibre (21.95 %) and a relatively small content of polyphenols (5.6 %) (Table 2). In the PTE preparation, the content of soluble fibre was lower (10.2 %), whereas the content of polyphenols considerably higher (28.8 %). Over 50 % of the dry matter of both preparations were carbohydrates (in the PTE extract without low-molecular carbohydrates) as well as crude ash and crude protein, particularly in the PTE preparation. The main group of polyphenols in the analysed strawberry extracts was represented by ellagic acid which, along with the ellagic acid bound with ellagitannins, constituted 90.4 % of total polyphenols of the PTW extract and 59.3 % of total polyphenols of the PTE preparation. In the PTW preparation, flavonols (mainly quercetin and kaempferol glycosides) and proanthocyanidins constituted additionally 4 and 3.6 % of total polyphenols, respectively. In the PTE preparation, second in terms of quantity was the group of proanthocyanidins (38 %), whereas flavonol aglycones (quercetin and kaempferol) and p-coumaric acid constituted the small remaining part.

**Table 2:** Chemical composition of polyphenolic preparations

	Phenolic preparations	
	PTW	PTE
Basic composition, g/100 g		
Dry matter	97.21	97.00
Crude ash	7.59	1.78
Crude protein	7.86	4.58
Ether extract	0.00	0.00
Dietary fibre (DF)	21.95	10.24
Low-molecular carbohydrates^a^	22.98	nd
Other carbohydrates	31.19	51.58
Polyphenolic compounds	5.64	28.82
Polyphenolic composition, g/100 g		
Free ellagic acid	0.40 (7.09)	1.20 (4.16)
Bound ellagic acid	4.70 (83.33)	15.90 (55.17)
Total ellagic acid	5.10 (90.43)	17.10 (59.33)
P-coumaric acid	0.01 (0.18)	0.02 (0.07)
P-coumaric acid derivative	0.00 (0.00)	0.07 (0.24)
Quercetin glucoside	0.01 (0.18)	0.03 (0.10)
Quercetin glucuronide	0.15 (2.66)	0.29 (1.01)
Kaempferol glucoside	0.05 (0.89)	0.11 (0.38)
Quercetin	0.01 (0.18)	0.20 (0.69)
Kaempferol	0.00 (0.00)	0.10 (0.35)
Anthocyanins	0.01 (0.18)	0.00 (0.00)
Proanthocyanidins (PC)	0.20 (3.55)	10.90 (37.80)

Values in brackets present per cent of individual component or a group to the total polyphenols

*PTW* strawberry polyphenols water extract, *PTE* strawberry polyphenols ethanol extract

^a^Saccharose, glucose and fructose, 10.33; 10.87 and 1.78, respectively, *PTE* strawberry polyphenols ethanol extract, *PTW* strawberry polyphenols water extract

In both preparations, the most important group of strawberry polyphenols was ellagitannins; however, in the water extract (PTW), the content of other polyphenols was at a minimal level, whereas in the water–methanol extract of a considerable contribution was polymerized proanthocyanidins. As it results from the calculation of the components of both pomace and the resultant PTW preparation, the yield of water extraction of polyphenols from pomace amounted to 36.7 %, including 24.5 % of ellagitannins. The application of the water–methanol extraction of pomace increased extraction performance of the total polyphenols to 59.5 %, including that of ellagitannins to 35.3 % and that of proanthocyanidins to 14 %.

### Animal growth and indices of gastrointestinal functioning

The applied dietary treatments had no effect on diet intake nor body weight gain of rats, but still they influenced some parameters of the gastrointestinal tract (Table 3). The addition of polyphenolic extracts to a diet increased the amount of caecal digesta, yet only in the case of the PTW extract was this amount statistically higher than in the control group (*P* = 0.001). In comparison with the rats receiving cellulose in their diets, the diets with FOS significantly increased the mass of the small intestine with digesta as well as the mass of caecal digesta (both *P* = 0.001). The addition of FOS to a diet resulted also in the reduction in digesta pH in the small intestine and in the caecum and in the reduction in dry matter content of caecal digesta (*P* = 0.001). A significant correlation (*P* = 0.007) was noted between the type of extract from strawberry pomace (PTW or PTE) and the type of dietary fibre (cellulose or FOS) only in the case of the mass of the small intestine with digesta. It was due to the fact that the addition of PTE preparation reduced the effect of FOS on the mass of the small intestine with digesta recorded in control groups and groups fed a diet with the PTW preparation.

**Table 3:** Diet intake (DI), body weight gain (BWG) and parameters of rat intestines development and functioning

	DI g/28 d	BWG g/28 d	Small intesine		Caecum			
			Weight, g/100 g of BW	pH	Content g/100 g of BW	Dry matter, %	Ammonia mg/100 g of digesta	pH
Treatment								
C_C_	489.8	120.4	3.07^c^	7.01	0.85	24.4	0.25	7.05
C_FOS_	491.7	122.7	3.45^ab^	6.68	1.34	18.1	0.23	6.64
PTW_C_	534.6	131.3	2.95^c^	7.03	1.24	22.7	0.26	7.03
PTW_FOS_	512.9	132.5	3.64^a^	6.79	1.66	16.5	0.26	6.82
PTE_C_	522.9	127.3	3.13^c^	6.98	1.14	22.5	0.24	7.02
PTE_FOS_	515.3	124.3	3.22^bc^	6.80	1.41	18.0	0.26	6.80
SEM	7.11	2.18	0.05	0.03	0.05	0.58	0.01	0.04
Phenolic (P)								
C	490.7	121.5	3.27	6.84	1.09^b^	21.3	0.24	6.84
PTW	523.8	131.9	3.30	6.91	1.45^a^	19.6	0.26	6.92
PTE	519.1	125.8	3.18	6.89	1.27^ab^	20.3	0.26	6.91
*P* value	0.134	0.169	0.380	0.624	0.001	0.264	0.432	0.599
Fibre (F)								
Cellulose	515.8	126.3	3.05^b^	7.00^a^	1.07^b^	23.2^a^	0.25	7.03^a^
FOS	506.6	126.5	3.44^a^	6.76^b^	1.47^a^	17.5^b^	0.25	6.76^b^
*P* value	0.523	0.969	0.001	0.001	0.001	0.001	0.752	0.000
Interaction P × F	0.792	0.876	0.007	0.578	0.484	0.623	0.536	0.422

*DI* diet intake, *BWG* body weight gain, *BW* body weight, *C*_*C*_ control diet with cellulose, *C*_*FOS*_ control diet with fructooligosaccharides, *PTE*_*C*_ diet with strawberry polyphenols ethanol extract with cellulose, *PTE*_*FOS*_ diet with strawberry polyphenols ethanol extract with fructooligosaccharides, *PTW*_*C*_ diet with strawberry polyphenols water extract with cellulose, *PTW*_*FOS*_ diet with strawberry polyphenols water extract with fructooligosaccharides, *PTW* strawberry polyphenols water extract, *PTE* strawberry polyphenols ethanol extract, *C* cellulose, *FOS* fructooligosaccharides

Values in columns marked with different letters are statistically different at p < 0.05

#### Glycolytic activity of microflora and the concentration of short-chain fatty acids

**Fig. 1:**
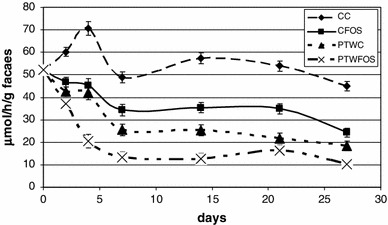
Activity of faecal β-glucuronidase of PTW preparation compared to control groups. *C*_*C*_ control diet with cellulose, *C*_*FOS*_ control diet with fructooligosaccharides, *PTW*_*C*_ diet with strawberry polyphenols water extract with cellulose, *PTW*_*FOS*_ diet with strawberry polyphenols water extract with fructooligosaccharides

**Fig. 2:**
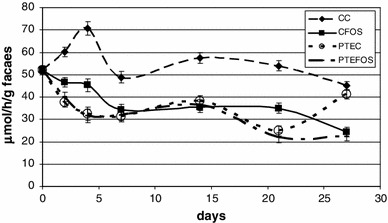
Activity of faecal β-glucuronidase of PTE preparation compared to control groups. *C*_*C*_ control diet with cellulose, *C*_*FOS*_ control diet with fructooligosaccharides, *PTE*_*C*_ diet with strawberry polyphenols ethanol extract with cellulose, *PTE*_*FOS*_ diet with strawberry polyphenols ethanol extract with fructooligosaccharides

The administration of the compared sources of strawberry polyphenols in a diet in the dose of 0.6 g of ellagic acid/kg diversified the activity of bacterial β-glucosidase and β-glucuronidase in faeces of the rats (Table 4, Figs. 1 and 2). In the rats receiving diets containing both polyphenolic extracts, the activity of β-glucosidase in faeces was significantly lower in all the analysed periods. The content of FOS in a diet suppressed the activity of β-glucosidase, however, only on the 28th day of the experiment was the difference statistically significant compared to the diet with cellulose (*P* = 0.001). A statistically significant effect of FOS on the activity of β-glucosidase was recorded only in the case of diets with PTE preparation. The applied dietary treatments had a diversified effect on the activity of β-glucosidase in rat faeces. Significant reductions in its activity were recorded in rats receiving diets with the PTW preparation. A similar effect of the PTE preparation was statistically significant only on days 7 and 21 of the experiment. A statistically significant correlation regarding the effect of both experimental factors (extracts from strawberry pomace and type of dietary fibre in a diet) on the activity of β-glucosidase was recorded in faeces of 28-week-old rats. This resulted from the fact that the PTE addition to a diet with FOS significantly reduced the activity of this enzyme in relation to the diet with cellulose, whilst PTW addition did not evoke such an effect. In the case of β-glucuronidase, an inverse tendency was reported. The concurrent application of PTW and FOS yielded a significant decrease in the activity of this enzyme, whereas PTE addition did not trigger such an effect.

**Table 4:** Activity of β-glucosidase and β-glucuronidase (μmol/h/g faeces) in rat faeces in the successive days of the experiment (0, 7, 14, 21 and 28)

	β-glucosidase (day)					β-glucuronidase (day)				
	0	7	14	21	28	0	7	14	21	28
Treatment										
C_C_	16.7	11.7	14.2	16.4	7.31^b^	52.2	48.9	57.4	53.9	45.0
C_FOS_		13.3	15.9	16.1	6.41^bc^		34.3	35.4	35.0	24.3
PTW_C_		9.89	10.3	9.53	4.53^cd^		25.5	25.4	21.8	18.5
PTW_FOS_		5.24	5.26	7.12	3.33^d^		13.3	12.6	16.4	10.4
PTE_C_		10.3	12.6	10.7	10.3^a^		31.3	38.1	24.9	41.2
PTE_FOS_		7.95	7.89	7.81	3.43^d^		31.9	36.4	21.8	22.4
SEM		0.73	0.89	0.94	0.43		2.16	2.64	2.31	2.19
Phenolic (P)										
C		12.5^a^	15.1^a^	16.2^a^	6.86^a^		41.6^a^	46.4^a^	44.5^a^	34.7^a^
PTW		7.56^b^	7.77^b^	8.33^b^	3.93^b^		19.4^c^	19.0^b^	19.1^b^	14.4^b^
PTE		9.10^b^	10.2^b^	9.26^b^	6.85^a^		31.6^b^	37.2^a^	23.4^b^	31.8^a^
*P* value		0.013	0.001	0.001	0.001		0.001	0.001	0.001	0.001
Fibre (F)										
Cellulose		10.6	12.4	12.2	7.37^a^		35.2^a^	40.3^a^	33.5^a^	34.9^a^
FOS		8.83	9.68	10.3	4.39^b^		26.5^b^	28.2^b^	24.4^b^	19.0^b^
*P* value		0.194	0.080	0.256	0.001		0.009	0.002	0.004	0.001
Interaction P × F		0.163	0.135	0.787	0.001		0.128	0.096	0.078	0.132

*C*_*C*_ control diet with cellulose, *C*_*FOS*_ control diet with fructooligosaccharides, *PTE*_*C*_ diet with strawberry polyphenols ethanol extract with cellulose, *PTE*_*FOS*_ diet with strawberry polyphenols ethanol extract with fructooligosaccharides, *PTW*_*C*_ diet with strawberry polyphenols water extract with cellulose, *PTW*_*FOS*_ diet with strawberry polyphenols water extract with fructooligosaccharides, *PTW* strawberry polyphenols water extract, *PTE* strawberry polyphenols ethanol extract, *C* cellulose, *FOS* fructooligosaccharides

Values in columns marked with different letters are statistically different at p < 0.05

The applied dietary treatments decreased the activity of caecal microflora enzymes of rats (Table 5). The results of a two-way anova indicate that both extracts from strawberry pomace reduced the activity of α-galactosidase and β-glucuronidase (*P* = 0.001). The activity of both of these enzymes and of β-glucosidase was lower in the ceacal digesta of rats receiving diets with FOS. Only in the case of β-glucuronidase, a close to statistical significance interaction was recorded in the effect of strawberry pomace extracts and dietary fibre (*P* = 0.081), which resulted from the fact that the PTW preparation intensified the inhibition of this enzyme activity by FOS to a greater extent than the PTE preparation did.

**Table 5:** Activity of microbial enzymes in caecal digesta of rats μmol/h/g

	Glucosidase		Galactosidase		β-glucuronidase
	*α*	*β*	*α*	*β*	
Treatment					
C_C_	7.17	2.41	5.84	4.58	18.8
C_FOS_	5.93	1.54	4.72	5.44	9.28
PTW_C_	6.21	1.80	3.88	4.93	9.22
PTW_FOS_	5.46	1.43	3.02	5.43	5.96
PTE_C_	7.47	1.75	3.74	4.23	10.5
PTE_FOS_	6.88	1.22	2.52	4.52	7.86
SEM	0.32	0.10	0.27	0.30	0.87
Phenolic (P)					
C	6.55	1.97	5.28^a^	5.01	14.0^a^
PTW	5.84	1.61	3.45^b^	5.18	7.59^b^
PTE	7.18	1.49	3.13^b^	4.37	9.20^b^
*P* value	0.239	0.087	0.001	0.547	0.001
Fibre (F)					
Cellulose	6.95	1.99^a^	4.48^a^	4.58	12.9^a^
FOS	6.09	1.40^b^	3.42^b^	5.13	7.70^b^
*P* value	0.181	0.002	0.026	0.385	0.001
Interaction P × F	0.909	0.524	0.947	0.932	0.081

*C*_*C*_ control diet with cellulose, *C*_*FOS*_ control diet with fructooligosaccharides, *PTE*_*C*_ diet with strawberry polyphenols ethanol extract with cellulose, *PTE*_*FOS*_ diet with strawberry polyphenols ethanol extract with fructooligosaccharides, *PTW*_*C*_ diet with strawberry polyphenols water extract with cellulose, *PTW*_*FOS*_ diet with strawberry polyphenols water extract with fructooligosaccharides, *PTW* strawberry polyphenols water extract, *PTE* strawberry polyphenols ethanol extract, *C* cellulose, *FOS* fructooligosaccharides

Values in columns marked with different letters are statistically different at p < 0.05

The applied dietary treatments diversified also the concentration of short-chain fatty acids (SCFA) in caecal digesta of rats (Table 6). The results of the two-way anova indicate that in comparison with C diets, the diets with PTW and PTE significantly decreased the concentration of acetic acid, propionic and butyric acids. In rats receiving PTW and PTE in a diet, the concentration of the total SCFAs was significantly lower than in groups C (58.6 and 56.4 vs. 75.6 μmol/g, *P* = 0.001). Taking into consideration the differences in the amount of caecal digesta of rats, the total SCFAs content in the digesta was similar in rats from the control group and in those receiving the diet with PTW, whereas significantly lower in rats receiving the diet with PTE (83.7 and 86.1 vs. 71.4 μmol/100 g BW, *P* = 0.002). Due to the differences in the amount of caecal digesta, the pool of C4 and C5 fatty acids was similar and slightly changed due to the administration of the compared preparations.

**Table 6:** Concentration of short-chain fatty acids (SCFA) in ceacal digesta

	SCFA concentration, μmol/g							SCFA pool μmol/100 g BW
	C2	C3	C4i	C4	C5i	C5	Total	
Treatment								
C_C_	40.5	15.3^b^	1.20^a^	8.55	1.03^a^	0.93	67.5^b^	56.9^c^
C_FOS_	46.7	23.2^a^	0.28^d^	12.24	0.61^b^	0.75	83.7^a^	110.5^a^
PTW_C_	34.8	9.95^c^	0.56^bc^	5.44	0.74^ab^	0.92	52.4^c^	64.7^c^
PTW_FOS_	41.6	13.2^b^	0.76^b^	7.29	0.88^ab^	1.06	64.7^b^	107.4^a^
PTE_C_	31.1	13.6^b^	0.51^c^	7.39	0.90^ab^	0.73	58.7^bc^	67.0^c^
PTE_FOS_	31.1	13.7^b^	0.51^c^	11.8	0.70^ab^	0.69	54.1^c^	75.7^bc^
SEM	1.14	0.72	0.05	0.55	0.04	0.03	1.96	3.10
Phenolic (P)								
C	43.6^a^	19.2^a^	0.74^a^	10.4^a^	0.82	0.84^ab^	75.6^a^	83.7^ab^
PTW	38.2^b^	11.6^b^	0.66^ab^	6.36^b^	0.81	0.99^a^	58.6^b^	86.1^a^
PTE	31.1^c^	13.7^b^	0.51^b^	9.61^a^	0.80	0.71^b^	56.4^b^	71.4^b^
*P* value	0.001	0.001	0.009	0.001	0.985	0.001	0.001	0.002
Fibre (F)								
Cellulose	34.5^b^	13.0^b^	0.76^a^	7.39^b^	0.89	0.86	59.5^b^	63.6^b^
FOS	39.8^a^	16.7^a^	0.51^b^	10.5^a^	0.73	0.83	67.5^a^	95.1^a^
*P* value	0.011	0.001	0.001	0.001	0.080	0.620	0.004	0.001
Interaction P × F	0.182	0.002	0.001	0.471	0.047	0.085	0.005	0.002

*C*_*C*_ control diet with cellulose, *C*_*FOS*_ control diet with fructooligosaccharides, *PTE*_*C*_ diet with strawberry polyphenols ethanol extract with cellulose, *PTE*_*FOS*_ diet with strawberry polyphenols ethanol extract with fructooligosaccharides, *PTW*_*C*_ diet with strawberry polyphenols water extract with cellulose, *PTW*_*FOS*_ diet with strawberry polyphenols water extract with fructooligosaccharides, *PTW* strawberry polyphenols water extract, *PTE* strawberry polyphenols ethanol extract, *C* cellulose, *FOS* fructooligosaccharides

Values in columns marked with different letters are statistically different at p < 0.05

In relation to the diets with cellulose, the content of FOS increased the pool of SCFAs in caecal digesta of rats, which may be attributed to increased concentrations of acetic acid, propionic and butyric acids and a slightly reduced concentration of isobutyric acid induced by FOS. A statistically significant correlation was recorded regarding the effect of both experimental factors on the concentration of selected fatty acids, including propionic (*P* = 0.002), isobutyric (*P* = 0.001), isovaleric (*P* = 0.047) acids, the total SCFAs (*P* = 0.005) and the pool of SCFA (*P* = 0.002). In the majority of cases, this was due to the synergic effect of FOS and PTW preparation. In contrast, the PTE preparation was found not to affect the physiological effects of FOS. Relatively significant differences in the concentration of butyric acid in groups C, PTW and PTE were considerably smaller (11.3, 9.22 and 12.2 μmol/100 g BW, respectively) when pools of these acids were compared (data not presented). Likewise, some differences were noted in the concentration of other C4 and C5 fatty acids in the caecal digesta; however, the pool of the total sum of these acids was similar. The pool of C4i, C5i and C5 acids, defined as putrefactive SCFAs, amounted to 2.62, 3.56 and 2.59 μmol/100 g BW, respectively, for C, PTW and PTE groups.

## Discussion

The results of the presented study indicate that water extract polyphenols consisted in 90 % of ellagitannins, with a small contribution of flavonols, including proanthocyanidins. In comparison with the water extract, the ethanol extract contained over 5 times more polyphenols, whilst nearly 60 % of these were constituted by ellagitannins and almost the whole rest (38 % of the total of polyphenols) by polymerised proanthocyanidins. The determined differences resulted from both various effectiveness of extraction with water and alcohol and from polyphenols content in pomace, a material differing from the well-characterized strawberry fruit.

As it results from studies of many authors, contents of key polyphenols present in fresh strawberry fruits fits within a wide range, i.e. for free and bound ellagic acid—between 130 and 850 mg/kg of fresh fruits [30–32], respectively, for proanthocyanidins—from below 10 to more than 160 mg/kg [33, 34], for anthocyanins—from 124 to 600 mg/kg [30, 35], for coumaric acid—from 9 to 102 mg/kg [30, 36] and for flavonols—from 5 to 65 mg/kg [30–32]. The results obtained were found to depend on the variety and the origin of strawberry fruits as well as on the analytical methods applied [37]. For instance, with the thiol method applied, the determined content of proanthocyanidins accounted for up to 2.75 g/kg [38].

As it results from sparse analyses of the content and composition of strawberry pomace polyphenols, about half of the content of ellagitannins in processed strawberry fruits is to be found in juice and the rest remains in pomace, out of which 35 % in the pulp and ca. 15 % in the seeds [6, 30]. The content of ellagitannins in pomace amounts to 1,020–1,420 mg/100 g dm [6], whilst other pomace components include ellagic, p-coumaric, p-benzoic acids, quercetin, kaempferol and anthocyanins with contents of 95, 14, 4, 50, 60 and 30 mg/100 g dm, respectively [22]. The content of proanthocyanidins in strawberry pomace may reach even 2.5 % as they are bound with insoluble polysaccharides of the cell wall, which results in only a small part of these compounds being found in juices [38]. Also for this reason, the water extract obtained in the study contained a small amount of proanthocyanidins, whose content in the extract was increased by the use of alcohol. Moreover, proanthocyanidins are found in higher quantity in strawberry achenes [30], and the pomace used in the experiment was seedless. In proanthocyanidins of strawberries, (epi)catechin is the main building block, but propelargonidin [(epi)afzelechin] is also present (6–10 % of the proanthocyanidins) [34]. Because we used only (+)-catechin and (−)-epicatechin as standards for phloroglucinolysis we might have underestimated the content of proanthocyanidins.

In the presented experiment, the applied doses of polyphenolic extracts in a diet contribute an equivalent amount of 0.06 % of ellagic acid and a relatively small content of other components, crude protein and carbohydrates, including dietary fibre. Such an addition of polyphenols did not reduce diet intake nor body weight of experimental rats, but had an effect on the activity of some of the enzymes of intestinal microflora and its most important products. It is common knowledge that the physiological effect of polyphenols is determined by both the content of these compounds in a diet as well as their physiochemical properties, including the level of polymerization [15, 21]. As it results from the experiments of other authors, polymeric proanthocyanidins travel through the acidic environment of the stomach unchanged [39] and their considerable amount remains in the digesta of the lower section of the gastrointestinal tract [40]. Even with a high consumption of proanthocyanidins, relatively low, nanomolar concentration of their derivatives, proanthocyanidin dimers, was recorded in volunteers’ blood [41]. The increased excretion of phenolic acids with urine of volunteers consuming diets rich in proanthocyanidins indicates that these compounds are partially degraded by the microflora colonizing the gastrointestinal tract [42]. Worthy of notice is, however, that an increased content of these compounds might reduce the activity of digestive enzymes [41].

Polyphenolic extracts applied in the presented experiment reduced the activity of some enzymes of the intestinal microflora, above all of β-glucosidase and β-glucuronidase in caecal digesta and faeces. The microbial β-glucuronidase is acknowledged as a good marker of microflora deconjugating activity [43], whereas its reduced activity—as a beneficial phenomenon minimizing the risk of xenobiotics transformation into toxic substances which take part in the process of cancer induction [44]. The increase in the hydrolytic activity of β-glucosidase has both a negative and a positive meaning, as β-glucosidase participates in the synthesis of both toxins and anti-carcinogens [45].

Results obtained in the presented experiment indicate that the content of polyphenolic extracts from strawberry pomace in diets significantly reduced the activity of α-galactosidase and β-glucuronidase, which resulted in a reduced concentration of SCFAs in caecal digesta of rats. Results of other authors indicate that the increased content of polyphenols in a diet may inhibit the growth and reduce the abundance of gastrointestinal microflora [46, 47]. It was denoted that the flavonoid extract applied as a dietary supplement was to decrease the activity of bacterial β-glucosidase and β- and α-galactosidases in the caecal digesta of rats [47].

It was noted in the presented experiment that the water–alcohol extract, unlike the water extract, reduced the total production of SCFAs in caecal digesta. Such an effect should be explained by a higher content of dietary fibre and other carbohydrates in the water extract. In the experiment by Aprikian et al. [48], an apple pectin and a polyphenol-rich apple concentrate were more effective together than separately on caecal fermentation in rats. Similarly, in the experiment by Zdunczyk et al. [47], dietary addition of inulin to the flavonoid-diet increased activity of glycolytic enzymes of intestinal microflora, normalized hydration of digesta and significantly decreased the pH of caecal digesta.

The reported study showed also that the presence of FOS in a diet reduced the activity of microbial β-glucuronidase and simultaneously increased the production of SCFAs, including acetic acid, propionic and butyric acids in caecal digesta. This was consistent with findings of other authors from studies with fructooligosaccharides, oligosaccharides of other types or inulin [47, 49, 50]. As it results from a research by McBain and Macfarlane [51], the highest activity of β-glucuronidase is typical of *Escherichia coli* and *Clostridium*. Reduction in β-glucuronidase activity, without compromising other glycolytic enzymes, may be an indicator of favourable changes in the gut microflora populations in our experiment. One of the effects of the fermentation of insulin-type fructans in the large bowel is a selective process; bifidobacteria (and possibly a few other genera) are preferentially stimulated to grow, thus causing significant changes in the composition of the gut microflora by increasing the number of potentially health-promoting bacteria and reducing the number of potentially harmful species, including *E. coli* and *Clostridium* [49]. Larrosa et al. [52] found that ellagitannins from pomegranate and theirs metabolites, urolithins, decreased enterobacteria and increased lactobacilli and bifidobacteria. Moreover, both ellagitannins and urolithins decreased inflammation markers (iNOS, cycloxygenase-2, PTGES and PGE2) in colonic mucosa of rats. Giménez et al. [23] also shown that it is the urolithins, the ellagitannins metabolites, not ellagitannins themselves have an effect in preventing vascular inflammation. Other authors also found that the urolithins are responsible for serum and liver lipids reduction [20, 21].

In this context, beneficial should seem the synergic with FOS inhibition of β-glucuronidase activity in intestinal digesta and increased production of SCFA in rat caecum by the PTW preparation. It is common knowledge that one of the mechanisms by which the intestinal microbiota may reduce *Enterobacteriaceae* (including *Salmonella*) is the bacteriostatic effect of short-chain fatty acids in the caeca [53].

In conclusion, both the sublimation-dried water extract and the alcohol extract from strawberry pomace showed a similar effect on the enzymatic activity of intestinal microflora in rats. An important effect of the application of both extracts in a diet was the reduction in the activity of β-glucuronidase in caecal digesta and in faeces, indicating positive changes in the population of intestinal microflora. It was a similar effect to that obtained by substituting dietary cellulose with more easily fermentable FOS. A lower content of proanthocyanidins (3.6 % of the total polyphenols) and at the same time a higher content of the soluble carbohydrate fraction (together with dietary fibre constituting 76.1 % of the extract) in the water extract resulted in an increased production of SCFAs in caecal digesta, compared to the water–alcohol extract. The application of water extract from strawberry pomace in the diets intensified the physiological effects of FOS, e.g. in inhibiting the activity of β-glucuronidase and the production of SCFAs in caecum, whilst no such an effect was induced by the water–alcohol extract being richer in proanthocyanidins (38 % of the total polyphenols).

## Abbreviations

C_C_: Control diet with cellulose
C_FOS_: Control diet with fructooligosaccharides
FOS: Fructooligosaccharides
PTE: Strawberry polyphenols ethanol extract
PTE_C_: Diet with strawberry polyphenols ethanol extract with cellulose
PTE_FOS_: Diet with strawberry polyphenols ethanol extract with fructooligosaccharides
PTW: Strawberry polyphenols water extract
PTW_C_: Diet with strawberry polyphenols water extract with cellulose
PTW_FOS_: Diet with strawberry polyphenols water extract with fructooligosaccharides
SCFAs: Short-chain fatty acids
